# Human adipose tissue-resident monocytes exhibit an endothelial-like phenotype and display angiogenic properties

**DOI:** 10.1186/scrt438

**Published:** 2014-04-14

**Authors:** Amparo Navarro, Severiano Marín, Nicasia Riol, Francisco Carbonell-Uberos, María Dolores Miñana

**Affiliations:** 1 Regenerative Medicine Laboratory, Fundación Hospital General Universitario, 46014 Valencia, Spain; 2 Department of Plastic and Reconstructive Surgery, Consorcio Hospital General Universitario, Valencia, Spain; 3 Immunohematology Service, Centro de Transfusiones, Valencia, Spain

## Abstract

**Introduction:**

Adipose tissue has the unique property of expanding throughout adult life, and angiogenesis is required for its growth. However, endothelial progenitor cells contribute minimally to neovascularization. Because myeloid cells have proven to be angiogenic, and monocytes accumulate in expanding adipose tissue, they might contribute to vascularization.

**Methods:**

The stromal vascular fraction (SVF) cells from human adipose tissue were magnetically separated according to CD45 or CD14 expression. Adipose-derived mesenchymal stromal cells (MSCs) were obtained from SVF CD45^-^ cells. CD14^+^ monocytes were isolated from peripheral blood (PB) mononuclear cells and then cultured with SVF-derived MSCs. Freshly isolated or cultured cells were characterized with flow cytometry; the conditioned media were analyzed for the angiogenic growth factors, angiopoietin-2 (Ang-2), vascular endothelial growth factor (VEGF), basic fibroblast growth factor (bFGF), hepatocyte growth factor (HGF), granulocyte colony-stimulating factor (G-CSF), and granulocyte macrophage colony-stimulating factor (GM-CSF) with Luminex Technology; their angiogenic capacity was determined in an *in vivo* gelatinous protein mixture (Matrigel) plug angiogenesis assay.

**Results:**

CD45^+^ hematopoietic cells within the SVF contain CD14^+^ cells that co-express the CD34 progenitor marker and the endothelial cell antigens VEGF receptor 2 (VEGFR2/KDR), VEGFR1/Flt1, and Tie2. Co-culture experiments showed that SVF-derived MSCs promoted the acquisition of KDR and Tie-2 in PB monocytes. MSCs secreted significant amounts of Ang-2 and HGF, but minimal amounts of bFGF, G-CSF, or GM-CSF, whereas the opposite was observed for SVF CD14^+^ cells.

Additionally, SVF CD14^+^ cells secreted significantly higher levels of VEGF and bFGF than did MSCs. Culture supernatants of PB monocytes cultured with MSCs contained significantly higher concentrations of VEGF, HGF, G-CSF, and GM-CSF than did the supernatants from cultures without MSCs. Quantitative analysis of angiogenesis at 14 days after implantation demonstrated that neovascularization of the implants containing SVF CD14^+^ cells or PB monocytes previously co-cultured with MSCs was 3.5 or 2 times higher than that observed in the implants with SVF-derived MSCs. Moreover, immunofluorescence of Matrigel sections revealed that SVF CD14^+^ cells differentiated into endothelial cells and contributed to vascular endothelium.

**Conclusions:**

The results from this study suggest that adipose tissue-resident monocytes should contribute to tissue vascularization. Because SVF CD14^+^ cells were more efficient in inducing angiogenesis than SVF-derived MSCs, and differentiated into vascular endothelial cells, they may constitute a new cell source for cell-based therapeutic angiogenesis.

## Introduction

Adipose tissue is composed of mature adipocytes and a heterogeneous stromal cell population surrounding them termed stromal vascular fraction (SVF) [1]. Adipose tissue is highly vascularized and has the unique property of expanding and regressing throughout adult life [2]. Although angiogenesis is required in growing adipose tissue, [3] the mechanisms of adipose vascularization are not fully understood. In past years, it has become evident that in addition to endothelial cell sprouting from preexisting blood vessels, endothelial progenitor cells and/or myeloid cells contribute to the angiogenic process [4,5]. However, in an *in vivo* adipogenesis model, endothelial progenitor cells minimally contributed to new vasculature, although the role of myeloid cells in adipose-tissue angiogenesis was not investigated [6].

Adipogenesis and angiogenesis are two closely related processes during embryogenesis and in adult life [2,7,8], and accumulating evidence demonstrates a functional link among endothelial cells, adipocytes, and monocytes. Conditioned media from adipose endothelial cells increases preadipocyte proliferation rates [9], whereas inhibition of vascular endothelial growth factor (VEGF)-VEGF receptor 2 (VEGFR2) signaling reduces angiogenesis and inhibits adipocyte differentiation [10]. Conditioned media from mature adipocytes induce overexpression of the intracellular adhesion molecule and platelet/endothelial cell adhesion molecule (PECAM)-1 in adipose endothelial cells, which in turn increases blood monocyte adhesion and migration to/through endothelial cells [11]. Moreover, mature adipocytes expressing monocyte chemoattractant protein-1, interleukin-8, and leptin are directly involved in this chemotactic effect [11]. Leptin, which is elevated in the plasma of obese subjects [12], increases blood monocyte adhesion and transmigration in a concentration-dependent manner [11] and also induces angiogenesis [13].

In expanding adipose tissue, monocytes accumulate proportional to the body mass index and adipocyte area [11,14]; therefore, it is tempting to speculate that monocytes may contribute to adipose tissue growth. Most efforts have been directed to the study of monocytes/macrophages in the context of obesity [14-16]; therefore, little information exists regarding their possible angiogenic properties.

Therefore, to address this question, we characterized CD14^+^ cells contained in the SVF from human adipose tissue and determined the ability of these tissue-resident monocytes to induce angiogenesis in an *in vivo* Matrigel plug assay. In this study, mesenchymal stromal cells (MSCs) derived from adipose SVF, known to be angiogenic in animal models of ischemia [17,18], the nonhematopoietic CD45^-^ cell component of the SVF, and blood CD14^+^ monocytes were used as controls in these investigations.

## Methods

### Tissue sampling

Human adipose tissue was obtained from female patients undergoing abdominal lipectomy at the University General Hospital of Valencia (Table 1 summarizes the clinical and laboratory characteristics of patients). Peripheral blood (PB) was obtained from adult healthy donors. The study was approved by the Clinical Research Ethics Committee of the Valencia University General Hospital, and patients and donors signed an informed consent.

**Table 1:** Clinical characteristics of the study patients

**Variables**	**<45 years (*n*** **= 12)**	**≥45 years (*n*** **= 18)**
**Sociodemographics**		
Age (years)	34.7 ± 5.3 (24–39)	53.7 ± 5.1 (46–64)
White (%)	100	100
**Habits**		
Smoking (%)	33.3	50
Alcohol consumption (%)	8.3	0
Drugs consumption (%)	0	0
**Clinical characteristics**		
Glucemia (mg/dl)	94.75 ± 13.87 (67–108)	101.65 ± 28.50 (79–183)
Systolic BP (mm Hg)	128.33 ± 11.29 (111–138)	128.59 ± 21.72 (104–180)
Diastolic BP (mm Hg)	71.58 ± 9.10 (59–89)	70.53 ± 7.06 (58–82)
Diabetes (%)	0	16.7
Hypertension (%)	8.3	5.6
Hyperlipidemia (%)	0	5.6
Diabetes treatment (%)	0	16.7
Antihypertensive treatment (%)	8.3	5.6
Lipid-lowering treatment (%)	0	5.6

BP, blood pressure. Values are expressed as mean ± SD, or as percentages, when indicated. The range of values is given in parentheses.

### Cell isolation and culture

Human adipose tissue SVF was obtained after enzymatic tissue digestion with collagenase, as previously described [19]. The SVF cells were magnetically labeled with anti-CD45 or anti-CD14 microbeads (Miltenyi Biotec, GmbH, Bergisch Gladbach, Germany), and cell populations were separated by using the MACS system (Miltenyi Biotec). Adipose-derived MSCs were obtained from the SVF CD45^-^ isolated cells. In brief, CD45^-^ cells were plated at a density of 30,000 cells/cm^2^ in endothelial basal medium (EBM-2) supplemented with EGM-2MV SingleQuots containing VEGF, basic fibroblast growth factor (bFGF), insulin-like growth factor-1, epidermal growth factor, and 5% FBS, (Lonza Walkersville, Inc., Walkersville, MD, USA). After reaching 80% to 90% confluence, adherent cells were detached by using 0.25% trypsin-ethylenediaminetetraacetic acid (EDTA) solution (Lonza) and reseeded at the same density. All of the studies were performed at the end of the second passage. PB mononuclear cells were obtained by centrifugation over Ficoll-Paque (Stem Cell Technologies SARL, Grenoble, France), and CD14^+^ monocytes were isolated by positive selection by using anti-CD14 microbeads and the MACS system (Miltenyi Biotec). CD14^+^ cells were cultured in EGM-2MV with or without MSCs. In brief, MSCs were resuspended in EGM-2MV to a concentration of 0.75 × 10^6^ cells/ml and transferred to 24-well plates (100 μl/well). After 24 hours, 0.1 × 10^6^ monocytes (1 × 10^6^ cells/ml) were plated into the well (full-contact) or onto the membrane (0.4-μm pore size) of transwell cell-culture inserts (noncontact). At the indicated time points, monocytes were removed from the membrane by washing with phosphate-buffered saline (PBS) containing 1 m*M* EDTA or harvested together with MSCs by scraping and then analyzed with flow cytometry. All of the cell cultures were incubated at 37°C in a humidified 5% CO_2_ atmosphere.

### Generation of cells-conditioned media

PB mononuclear cells were divided in two parts; one half was used to isolate untouched monocytes by using an indirect magnetic labeling (Monocyte Isolation Kit II; Miltenyi Biotec), whereas the other half was used to obtain monocytes by positive selection. Each of the selected monocyte populations was cultured for 72 hours in RPMI (Lonza) with 5% autologous plasma (condition 1) or in EBM-2 with 2.5% FBS (condition 2). Additionally, monocytes were cultured in EGM-2MV (condition 3) or in a transwell system with adipose-derived MSCs for 5 days. At the end of the culture period, monocytes were switched to EBM-2 with 2.5% FBS, and 72 hours later, conditioned media were collected. Isolated SVF CD14^+^ cells were divided in two parts: one was cultured as in condition 2, and the remaining cells, as in condition 3. Adipose-derived MSCs were grown in EGM-2MV until confluent, and then switched to EBM-2 with 2.5% FBS. Seventy-two hours later, conditioned media were collected, centrifuged, and frozen at -80°C until use. Conditioned media were analyzed for the angiogenic or antiapoptotic growth factors, angiopoietin-2 (Ang-2), VEGF, bFGF, granulocyte colony-stimulating factor (G-CSF), granulocyte macrophage colony-stimulating factor (GM-CSF), and hepatocyte growth factor (HGF) with Luminex xMAP Technology by using a Luminex Screening Assay (R&D Systems Minneapolis, MN, USA).

### Flow-cytometry analysis

Cells were stained in PBS containing 1 m*M* EDTA and 1% bovine serum albumin (BSA) with specific antibodies or isotype-matched controls for 30 minutes at 4°C in the dark. After washing, cells were analyzed on a FACSCanto II (BD Biosciences, Erembodegem, Belgium) by using FACSDiva software (BD Biosciences). The antibodies used were CD9, CD13, CD14, CD31, CD34, CD45, CD144, CD146, CD90, CD105 (BD Biosciences), VEGFR1/Flt-1 (clone 49560; R&D Systems, Abingdon, UK), VEGFR2/KDR (clone ES8-20E6 from Miltenyi Biotec and clone 89106 from R&D Systems), and Tie2/Tek (clone 83715 from R&D Systems and clone 33.1(Ab33) from BioLegend, Uithoorn, The Netherlands) conjugated to fluorescein-isothiocyanate (FITC), phycoerythrin (PE), allophycocyanin (APC), and PE-cyanin7 (PE-Cy7) or APC-cyanin7 (APC-Cy7). 7-Amino-actinomycin D (Sigma-Aldrich, Madrid, Spain) was used for removal of nonviable cells in the analysis.

### *In vivo* Matrigel plug assay

Freshly isolated SVF CD45^+^, CD45^-^, or CD14^+^ cells, adipose-derived MSCs, and PB CD14^+^ monocytes, freshly isolated or cocultured with MSCs in a transwell system (1 × 10^6^ cells in 50 μl PBS) were individually mixed with 400 μl of growth-factor-reduced Matrigel (BD Biosciences) and injected subcutaneously into the dorsal flank of 8- to 12-week-old female Hsd:athymic nude-*Foxn1*^*nu*^ mice (Harlan Laboratories, Udine, Italy). Matrigel with PBS alone was used as negative control. Matrigel plugs were removed on day 14, fixed in zinc fixative solution (BD Biosciences), and embedded in paraffin for hematoxylin and eosin (H&E) staining or for immunohistochemistry. For the functional perfusion of neovessels in Matrigel, 10 minutes before the Matrigel plugs were harvested, FITC-dextran (MW 70,000, Sigma-Aldrich) was injected intravenously into the tail vein. The mice were killed by intraperitoneal sodium pentobarbital injection. All animal procedures were conducted in conformity with institutional guidelines in compliance with the Spanish guidelines for animal care (RD 1201/2005) and the European Community Council Directive (2010/63/UE), and were approved by the Council of Agriculture of the Valencian Regional Government and by the internal Committee for Ethics and Animal Research.

### Matrigel-plug immunohistochemistry and immunofluorescence

Matrigel plugs in 5-μm deparaffined sections were reacted with primary rabbit polyclonal anti-human CD45 (1/1,000 dilution) or anti-endothelial nitric-oxide synthase (eNOS) (1/100 dilution) (Abcam, Cambridge, UK), or monoclonal rat anti-mouse CD31 (1/25 dilution, clone MEC13.3; BD Pharmingen). For CD45 and e-NOS, antigens were heat-retrieved in Antigen Retrieval Solution (Dako, Glostrup, Denmark), and endogenous peroxidase activity blocked by using 3% hydrogen peroxide in methanol. Sections were incubated at 4°C overnight with the primary antibodies. Nonspecific protein-binding sites and endogenous mouse IgG were sequentially blocked by incubation in PBS containing 0.1% Triton X-100, 0.5% BSA, and 10% fetal bovine serum (FBS) for 1 hour at room temperature (RT) followed by nonconjugated AffiniPure Fab Fragment goat anti-mouse IgGs (Jackson ImmunoResearch Laboratories, West Grove, PA, USA) for 2 hours at RT. Sections were then incubated with biotin-conjugated secondary antibodies and treated with streptavidin-horseradish peroxidase, and the reaction was developed by using 3,3′-diaminobenzidine (DAB) substrate (LSAB + System-HRP; Dako).

For CD31, after blocking of endogenous peroxidase activity, the sections were incubated with the primary antibody at RT for 1 hour followed by blocking of nonspecific protein and endogenous mouse IgG. Primary antibodies were detected by sequential incubation with biotinylated secondary antibodies, streptavidin-horseradish peroxidase, and DAB substrate (Anti-Ig HRP Detection Kit, BD Biosciences). All of the slides were counterstained with hematoxylin.

For double immunofluorescence, tissue sections were dewaxed, rehydrated, incubated in Antigen Retrieval Solution (BD Pharmingen), permeabilized with 0.2% Triton-X100 in PBS for 30 minutes, and blocked for 1 hour at RT with 1% BSA and 10% normal goat serum (Jackson Immunoresearch Laboratories) in PBS. Sections were then immunostained with rabbit anti-human von Willebrand factor (vWF; 1/100 dilution, Sigma), rabbit anti-human nestin (1/250 dilution, Millipore, Temecula, CA, USA) or rabbit anti-α-smooth muscle actin (αSMA; 1/100 dilution; Abcam), overnight at 4°C, followed by goat anti-rabbit conjugated with Alexa 555 (Jackson Immunoresearch Laboratories). Then, to identify murine endothelial cells, slides were stained with rat anti-mouse CD31 (1/25 dilution, clone MEC13.3; BD Pharmingen) followed by goat anti-rat conjugated with Alexa 488.

To identify human endothelial cells, slides were blocked with Carbo-Free blocking solution (Vector Laboratories, Burlingame, CA, USA) and stained with biotinylated *Ulex europaeus* agglutinin 1 (UEA-1; 1/100 dilution, Vector Laboratories) for 1 hour at RT followed by fluorescein (DTAF)-conjugated streptavidin (Jackson Immunoresearch Laboratories). The αSMA and the vWF antibodies are cross-reactive for mouse and human. Images were acquired by using a Leica DFC480 camera and Leica DM6000 microscope with the Leica Application Suite (LAS), Version 3.6.0.

### Neovessel quantification in Matrigel plugs

In H&E-stained sections, newly formed vessels were defined as endothelium-lined tubular structures containing clearly identifiable erythrocytes. One in every five slides was counted, by using a 40× objective, in a blinded fashion, and the number of vessels per square millimeter was averaged. Five animals were used for each condition. The lumen area was determined by using Leica Application Suite, v4.0 software (Leica Microsystems, Wetzlar, Germany).

### Statistical analysis

The results were expressed as the mean ± standard deviation. The data were analyzed by using GraphPad Prism Software 5.0 (GraphPad Software Inc., La Jolla, CA, USA). The comparisons among groups were analyzed in a one-way ANOVA followed by Bonferroni *post hoc* test and, when appropriate, by Student *t* test. Differences were considered to be statistically significant at *P* < 0.05.

## Results

### Hematopoietic cell population from the SVF contains CD14^**+**^ cells expressing CD34 and displaying an endothelium-like phenotype

**Figure 1:**
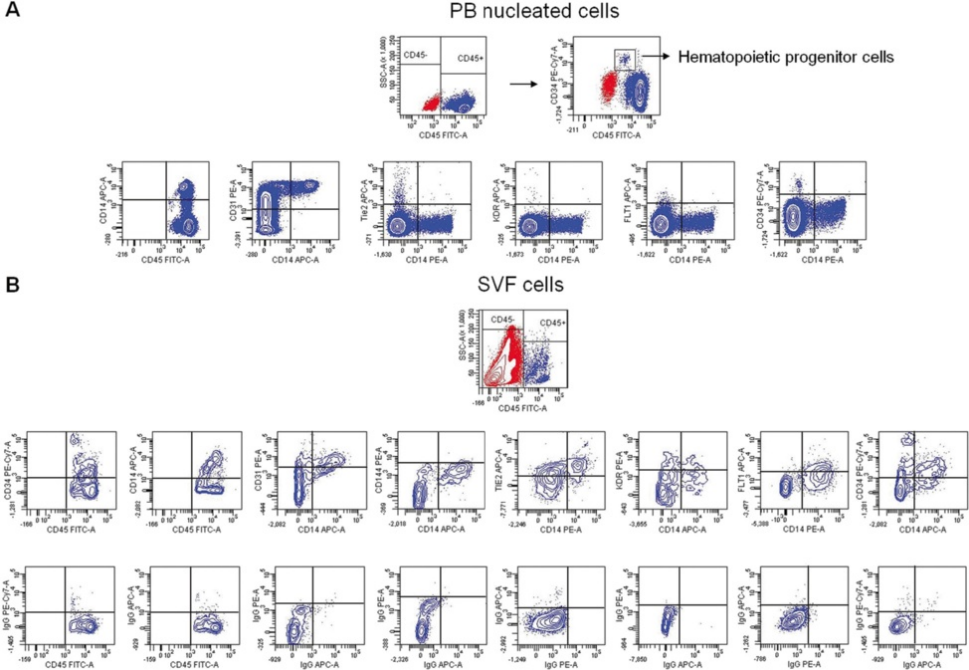
**SVF CD14**^**+ **^**cells exhibit a proangiogenic phenotype.** PB nucleated cells **(A)** were analyzed for CD45 and CD34 expression. Top right, CD34^+^CD45^low^ cells corresponding to hematopoietic progenitor cells are shown. Bottom, CD45^+^ cells (blue) were gated and analyzed for CD14 expression and for co-expression of CD14 with CD31, Tie2, KDR, Flt-1, and CD34. The CD14^+^ cells represented 20% of CD45^+^ cells, which in turn accounted for 98.5% of the total viable PB nucleated cells. SVF cells **(B)** were analyzed for CD45 expression, and then the gated CD45^+^ cells (blue) were analyzed for CD34 and CD14 expression and for the co-expression of CD14 with CD31, CD144, Tie2, KDR, Flt-1, and CD34. Isotype-matched controls are shown. CD45^+^ cells accounted for 6% of total viable cells. Dot plots of CD14 conjugated with APC or PE correspond to three different samples.

Human adipose tissue SVF reportedly contains a variable proportion of CD34^+^ cells [1,19,20]. Here, we demonstrate that CD34^+^ cells accounted for approximately 50% of SVF cells. Most CD34^+^ cells expressed CD90 and CD13, which was indicative of their mesodermal origin, and a lower proportion were positive for CD31 (Table 2). As expected, CD45^-^ cells were abundant in the SVF, and the majority of these cells expressed CD34. Unexpectedly, we found that approximately 30% of the remaining CD45^+^ hematopoietic cells also expressed CD34 (Table 2). In human PB, CD34 is a marker of hematopoietic progenitors; thus, virtually all CD34^+^ cells exhibit little or no CD45 expression, lack expression of cell-surface markers that are associated with the CD45^+^ lineage [21] and are clustered in a discrete cell population when analyzed with flow cytometry (Figure 1A). However, approximately 65% of CD45^+^CD34^+^ SVF cells co-expressed CD14 (Table 2; Figure 1B). Further analysis of the CD45^+^ SVF cells demonstrated that 35% of them expressed CD14, of which 55% co-expressed CD34 (Figure 1B), whereas most of the remaining CD45^+^ cells identified the lymphocyte subset. Remarkably, most adipose CD14^+^ cells expressed CD31 and exhibited an endothelium-like phenotype, in contrast to what was observed in circulating CD14^+^ monocytes, as demonstrated by KDR, Tie-2, and Flt1 co-expression (Table 2, Figure 1B), although CD144 expression was barely detected (Figure 1).

**Table 2:** Surface marker expression on freshly isolated SVF cells

**Cells**	**Percentage mean ± SD**
Crude SVF cells	
CD45	19 ± 11 (*n* = 30)
CD34	48 ± 20 (*n* = 30)
SVF CD45^-^ cells	
CD34	70 ± 20 (*n* = 30)
SVF CD45^+^ cells	
CD34	29 ± 13 (*n* = 30)
CD14	35 ± 13 (*n* = 20)
SVF CD34^+^ cells	
CD90	90 ± 9 (*n* = 20)
CD13	77 ± 17 (*n* = 10)
CD31	22 ± 11 (*n* = 20)
SVF CD45^+^CD34^+^ cells	
CD14	64 ± 17 (*n* = 12)
SVF CD45^+^CD14^+^ cells	
CD34	55 ± 13 (*n* = 20)
CD31	66 ± 16 (*n* = 10)
KDR	55 ± 17 (*n* = 8)
Tie-2	32 ± 14 (*n* = 8)
Flt-1	34 ± 23 (*n* = 5)

Cell populations in the stromal vascular fraction were characterized with flow cytometry. Values indicate the percentage of cells expressing selected antigens within the SVF cell subsets. Results are expressed as mean ± SD.

### Adipose-derived MSCs promote the expression of endothelial markers in circulating monocytes

**Figure 2:**
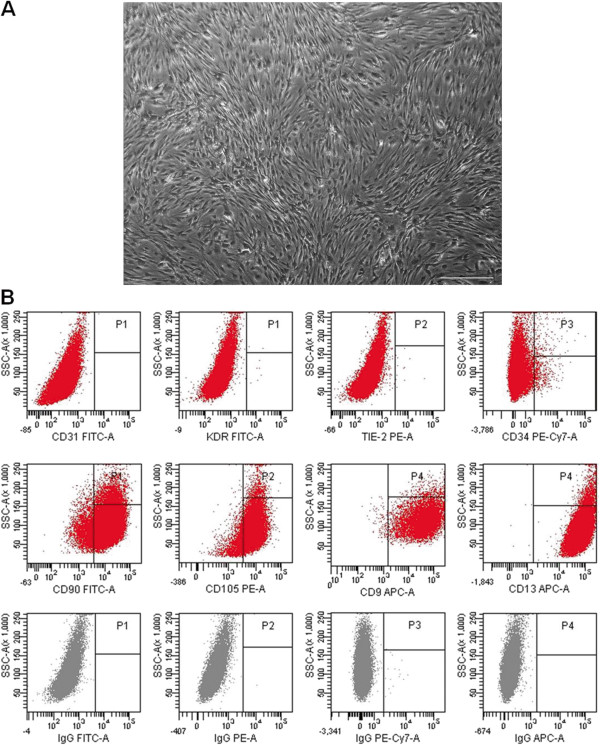
**Phenotypic characteristics of adipose-derived MSCs.** CD45^-^ SVF cells were isolated by immunomagnetic methods and then cultured in EGM-2 MV to generate MSCs. Morphologic aspect of MSCs at passage 2 (scale bar, 100 μm) is shown in panel **(A)**. Flow-cytometry dot plots demonstrating the expression of a panel of markers in MSCs are shown in **(B)**. Isotype-matched controls are given.

**Figure 3:**
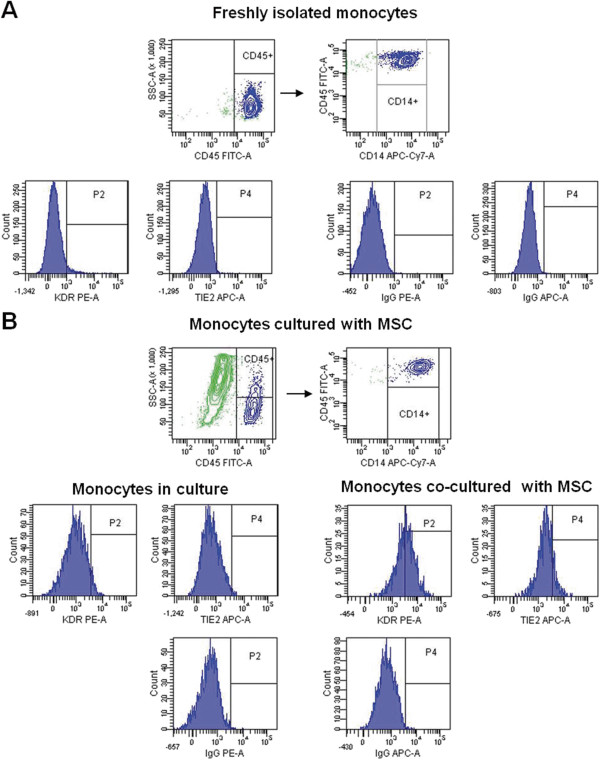
**Induction of endothelial markers in circulating CD14**^**+ **^**cells.** CD14^+^ monocytes were isolated from adult PB, cultured in EGM-2 MV with or without MSCs, and then analyzed with flow cytometry. Dot plots from freshly isolated CD14^+^ cells **(A)** and after 4 days of co-culture with adipose-derived MSCs (green) **(B)** are shown. Flow-cytometry histograms in panels A and B show the expression of KDR and Tie2 in gated CD14^+^ cells. Isotype-matched controls are given.

Because adipose stromal cells secrete a wide variety of angiogenic factors [22], circulating CD14^+^ monocytes could acquire an endothelial cell phenotype after tissue recruitment. To test this hypothesis, PB CD14^+^ cells were cultured on a monolayer of adipose-derived MSCs, and the induced phenotypic changes were evaluated. As demonstrated in Figure 2A, SVF-derived MSCs had typical fibroblast morphology; expressed the mesodermal antigens CD9, CD13, CD90 and CD105; and lacked expression of CD31, CD34, VEGFR2/KDR and Tie-2 (Figure 2B). We observed that after 3 days in co-culture, approximately 40% (range, 20% to 50%; *n* = 4) and 30% (range, 10% to 40%; *n* = 4) of CD14^+^ monocytes expressed KDR and Tie-2, respectively (Figure 3B), and these cell proportions were maintained for the next 4 days. Of note, a similar level of induction was observed when CD14^+^ monocytes were seeded into transwell membranes; thus, direct contact between monocytes and MSCs was not necessary to induce changes in their antigen-expression profile. As expected, no phenotypic changes were observed when CD14^+^ cells were cultured in endothelial growth medium, most likely because they were cultured on tissue-culture plastic (Figure 3B). However, CD34 expression was not induced in these culture conditions (data not shown).

### SVF CD14^+^ cells are highly angiogenic

**Figure 4:**
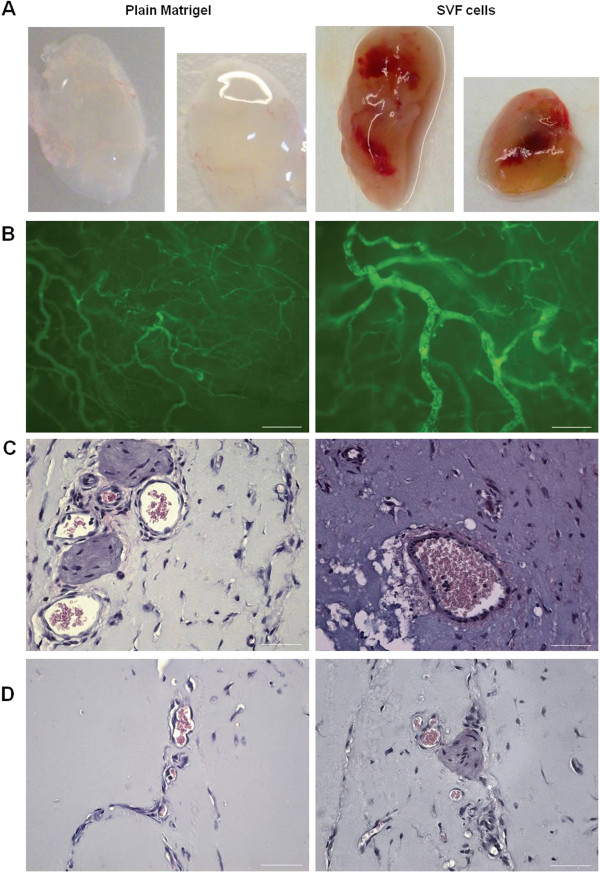
**Angiogenic response induced by SVF cells in the Matrigel-plug assay.** Freshly isolated SVF CD45^+^, CD45^-^or CD14^+^ cells, or SVF-derived MSCs were mixed with Matrigel and injected subcutaneously into immunodeficient mice. **(A)** Macroscopic visualization of Matrigel plugs containing SVF cells or no cells 14 days after implantation. In some experiments, 10 minutes before the Matrigel plugs were harvested, mice were injected into the tail vein with FITC-dextran. Fluorescence microscopy of Matrigel explants allows identifying vessels connected to the circulation **(B)**. Sections from the plugs were stained with H&E to visualize vessel formation **(C, D)**. Scale bars: B, 200 μm (left), 100 μm (right); C and D, 50 μm.

**Figure 5:**
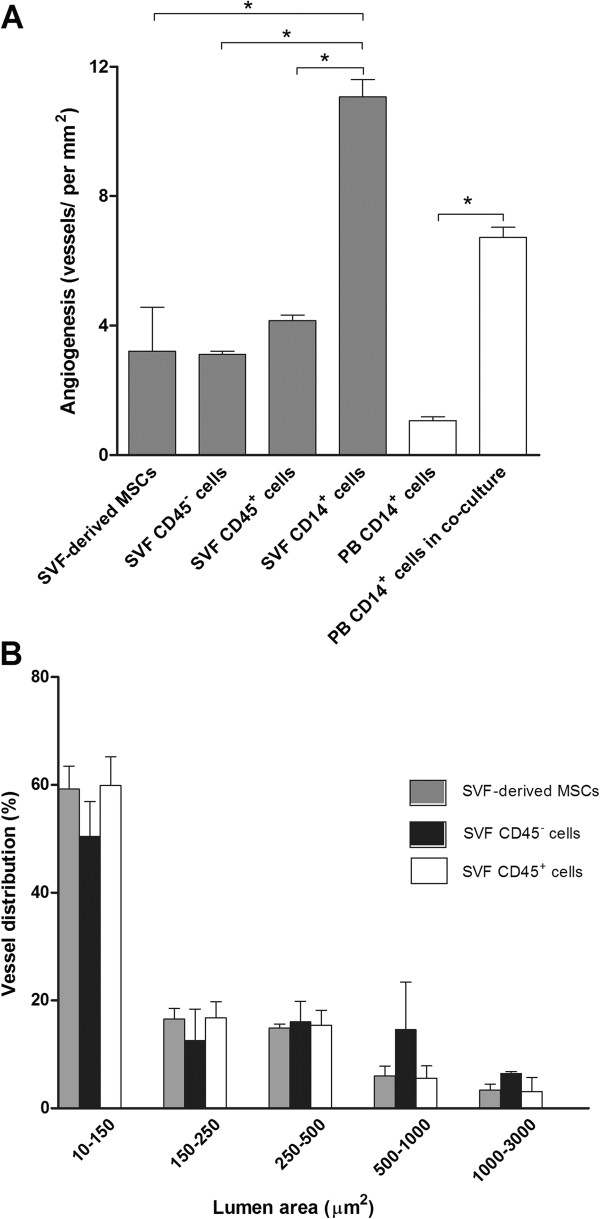
**Quantification of neovessels in Matrigel plugs.** Freshly isolated SVF cells, SVF-derived MSCs, and PB CD14^+^ monocytes, freshly isolated or after co-culture with SVF-derived MSCs in a transwell system, were individually embedded in Matrigel at 1 × 10^6^ cells per implant. Matrigel explants were harvested 14 days after implantation and H&E-stained sections were used to enumerate blood vessels **(A)**. The lumen area of newly formed vessels in Matrigel implants containing SVF-derived MSCs or SVF-isolated CD45^+^ or CD45^-^ cells was determined. Vessel distribution indicates the percentage of vessels according to their lumen area, given as range of values **(B)**. Results are expressed as the mean and SD of five Matrigel implants per test group. MSCs derived from three independent SVFs were used. *P < 0.0001 for differences between groups linked by the brackets.

To determine the angiogenic capacity of hematopoietic cells contained in the SVF, we performed subcutaneous Matrigel implantation with SVF CD45^+^ cells and compared the resulting angiogenesis with that obtained by using SVF CD45^-^ cells and SVF-derived MSCs. Two weeks after implantation, the gross morphologic appearance of the explanted plugs revealed the induction of angiogenesis in Matrigel implants containing SVF-derived cells in contrast to the lack of angiogenesis that was observed with Matrigel alone (Figure 4A). Macroscopically, the neovessels induced by SVF cells were well organized and formed a dense capillary network, and some blood vessels with the appearance of arterioles were observed (Figure 4B). Histologic sections demonstrated the presence of blood vessels lined with endothelial cells of very different sizes that contained varying amounts of erythrocytes (Figure 4C,D). Interestingly, the number of neovessels formed in implants containing SVF CD45^+^ cells was higher (1.3-fold increase) than in those containing SVF CD45^-^ cells or SVF-derived MSC, which was nearly identical (Figure 5A). Nevertheless, no differences in size distribution were observed among vessels induced by SVF CD45^+^ cells and those induced by SVF CD45^-^ cells or MSCs (Figure 5B). Although most Matrigel-implant neovessels were small vessels with a lumen area ≤150 μm^2^, we also observed the development of a few very large blood vessels (lumen area ranging between 1,000 and 3,000 μm^2^).

Given the cellular composition of the SVF CD45^+^ cell population, CD14^+^ cells must be the main cells responsible for the angiogenic activity observed. When isolated SVF CD14^+^ cells were injected, a robust angiogenesis was noticed; on a per-cell basis, the number of neovessels induced by SVF CD14^+^ cells was approximately 3.5-fold increased when compared with that induced by SVF-derived MSCs (Figure 5A). Finally, we tested whether the co-culture system led blood monocytes to increase their angiogenic capacity. As shown in Figure 5A, the number of neovessels in implants containing PB monocytes co-cultured with SVF-derived MSCs in a transwell system was 6 times higher than that obtained by using PB-isolated monocytes.

### SVF CD14^+^ cells secrete angiogenic factors

To explore further the possibility that SVF CD14^+^ cells and PB monocytes that had been cultured with MSC promoted angiogenesis in a paracrine manner, we determined the levels of the angiogenic and antiapoptotic factors Ang-2, VEGF, bFGF, G-CSF, GM-CSF, and HGF in the conditioned media. We found that SVF CD14^+^ cells cultured in EGM-2MV secreted approximately threefold more VEGF and up to 16-fold more bFGF than the SVF-derived MSCs cultured in the same medium. By contrast, a lesser secretion of Ang-2 was observed, and HGF was minimally secreted. Importantly, when SVF CD14^+^ cells were cultured in EBM-2, a medium that does not contain angiogenic factors, the level of secretion of VEGF and bFGF was increased approximately twofold, whereas that of G-CSF and GM-CSF was decreased by six- and twofold, respectively (Table 3). Next, to assess whether the type of selection used to isolate PB monocytes had affected their ability to secrete angiogenic factors, we obtained conditioned media from both positive selected monocytes and indirectly selected monocytes, termed untouched monocytes. As seen in Table 3, no differences were found in the secretion of growth factors studied between monocytes obtained by either of the two methods, in any of the culture media used. Nevertheless, untouched monocytes secreted higher amounts of G-CSF, and slightly lower levels of Ang-2, than positive selected monocytes when cultured in EBM-2 or EGM-2MV, respectively. When PB CD14^+^ cells were co-cultured with MSCs, they were induced to secrete high levels of VEGF, and of G-CSF and GM-CSF, reaching values similar to those observed in SVF-derived MSCs, and SVF CD14^+^ cells cultured in EGM-2MV, respectively.

**Table 3:** **Secretion of growth factors by SVF-derived MSC, SVF CD14**^**+ **^**cells, and PB monocytes**

	**Ang-2**	**VEGF**	**HGF**	**bFGF**	**G-CSF**	**GM-CSF**
**SVF-derived MSCs**	1,642 ± 1,157	329 ± 69	12,602 ± 1,492	21 ± 3	70 ± 13	3.2 ± 1.2
**PB-positive selected monocytes**						
Condition 1	200 ± 35	40 ± 5	7 ± 1	12 ± 5	0.6 ± 0.2	1.9 ± 0.2
Condition 2	186 ± 78	52 ± 25	20 ± 19	12 ± 2	7.4 ± 2.2	15 ± 4
Condition 3	278 ± 18	48 ± 15	137 ± 35	25 ± 2	3.4 ± 1.2	5 ± 2
Co-cultured with MSCs	26 ± 9*	210 ± 49	155 ± 71*	34 ± 4	4,188 ± 2,001*	957 ± 552*
**PB-untouched monocytes**						
Condition 1	229 ± 36	46 ± 36	9 ± 1	11 ± 2	0.7 ± 0.3	1.5 ± 0.7
Condition 2	215 ± 142	54 ± 43	28 ± 23	16 ± 6	33 ± 7^†^	18 ± 8
Condition 3	216 ± 25^†^	61 ± 16	163 ± 75	18 ± 3	4.7 ± 1.4	4.3 ± 1.7
Co-cultured with MSCs	25 ± 9*	197 ± 96	134 ± 47*	27 ± 5^†^	4,321 ± 2,529*	878 ± 514*
**SVF CD14**^**+ **^**cells**						
Condition 2	220 ± 123*	1,733 ± 193*	18 ± 8*	580 ± 199*	30,895 ± 6,699*	4,017 ± 791*
Condition 3	176 ± 96*^‡^	916 ± 164*^†^	13 ± 4*^‡^	341 ± 121*^†‡^	5,555 ± 3,986*^†^	1,782 ± 1,001*^†^

Cells derived from adipose tissue or PB monocytes were cultured as described in Methods, and the 72-hours conditioned media were analyzed by Luminex Screening Assays to determine the secretion of selected growth factors. Condition 1, RPMI with autologous plasma; condition 2, EBM-2 with FBS; and condition 3, EGM-2MV with FBS and then switched to EBM-2 with FBS. Values are expressed as mean ± SD pg of the secreted factor normalized to 10^6^ cells. SVF from six different donors were used to generate MSCs. SVF CD14^+^ cells were isolated from the same SVF samples; PB monocytes were obtained from four different donors, and were co-cultured with MSCs derived from four different donors. Statistical significance: SVF CD14^+^ cells or PB monocytes versus SVF-derived MSCs, *P < 0.0001; SVF CD14^+^ cells cultured in condition 2 versus SVF CD14^+^ cells cultured in condition 3, and PB-positive selected monocytes versus PB untouched monocytes: cultured in condition 2 or cultured in condition 3, ^†^*P* < 0.0001; SVF CD14^+^ cells versus PB monocytes co-cultured with MSCs, ^‡^*P* < 0.0001.

### SVF CD14^+^ cells incorporate into neovessels

**Figure 6:**
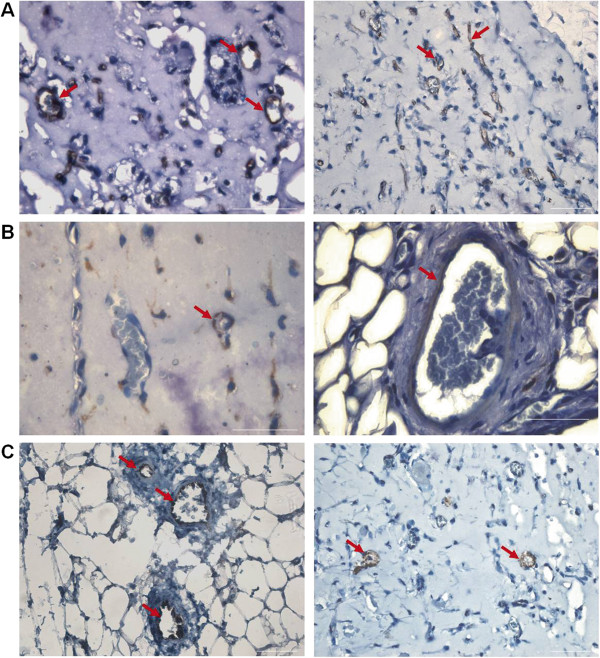
**SVF CD14**^**+ **^**cells incorporate into new vessels.** Fourteen days after implantation, Matrigel implants containing SVF CD14^+^ cells were evaluated for the expression of CD31, CD45, and e-NOS. **(A)** Representative images for CD31 staining. Arrows indicate the presence of CD31^+^ endothelial cells in the vessels formed. **(B)** Human CD45 immunostaining. Note that CD45^+^ cells are located surrounding blood vessels (left) or adjacent to the endothelium (right). **(C)** Human e-NOS immunostaining. Arrows indicate positive staining of some blood vessels for e-NOS. Scale bars: A, 50 μm; B, 50 μm (left), 30 μm (right); C, 50 μm.

Neovessel recruitment and formation into the Matrigel plugs with SVF cells were also evaluated by staining with anti-mouse CD31 antibody. Because nude mice were used for *in vivo* angiogenesis assay, the explanted plugs did not demonstrate inflammatory reactions, and cells expressing CD31 must correspond to host endothelial cells. The majority of the endothelial cells lined together and formed blood vessels (Figure 6A). To investigate the contribution of the SVF CD14^+^ cells to the establishment of a vascular network in Matrigel plugs, sections were stained with anti-human CD45. Figure 6B demonstrates that most CD45^+^ cells were localized near or surrounding blood vessels. However, in random Matrigel-explant sections, we noticed the presence of arterioles, as defined by their structure (presence of smooth muscle cells within the wall), that contained CD45^+^ cells adjacent to the endothelium (Figures 4C and 6B).

Last, sections were stained with anti-human e-NOS (Figure 6C). As expected, most vascular structures stained positive for e-NOS, and the pattern of staining was similar to that observed for CD45.

### SVF CD14^+^ cells differentiate into endothelial cells

**Figure 7:**
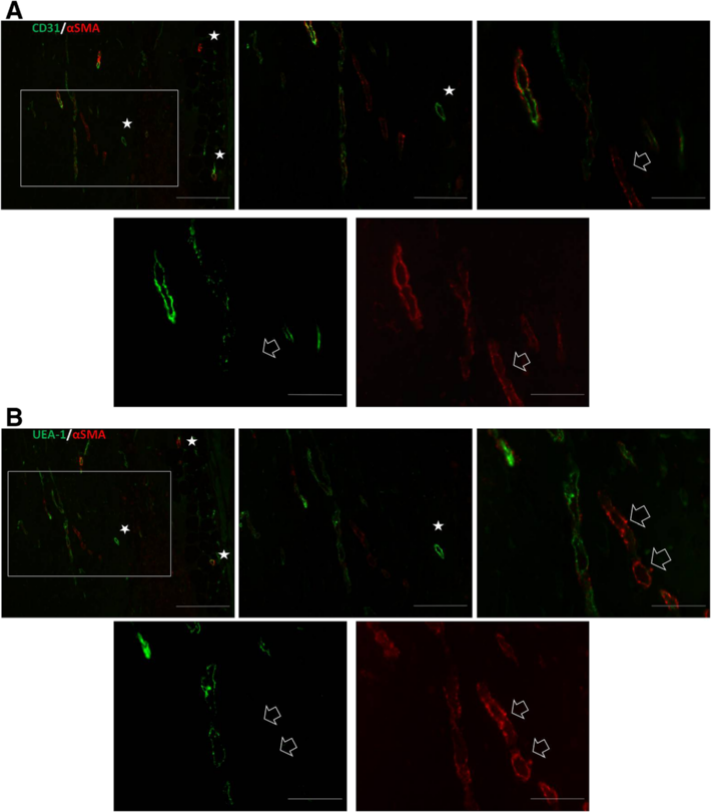
**Matrigel implants contain blood vessels comprising human and mouse endothelial cells.** Consecutive sections of Matrigel implants with SVF CD14^+^ cells were double-immunostained with antibodies against mCD31 (green) and αSMA (red) **(A)**, or *Ulex europaeus* agglutinin 1 (UEA-1; green) and αSMA (red) **(B)**. The images on the top of the panels A and B show merged images and correspond to the same microscopic field at different magnifications: left, low magnification; middle, medium magnification; right, higher magnification. Images on the bottom **(A, B)** show green and red fluorescence and correspond to the merged image at the highest magnification. Images in panel B correspond to a consecutive section. The insets in **A** and **B** (upper left) show the field at low magnification and asterisks illustrate benchmarks. Note that the green staining associated with murine **(A)** or human **(B)** endothelial cells in the adjacent sections is near identical. Double labeling shows a close assembly of αSMA-positive cells to the blood vessels. White arrows indicate αSMA-positive cells lining vascular-like structures which do not express murine **(A)** or human **(B)** endothelial cells. Scale bars in A, B (upper left): 200 μm, upper in the middle: 100 μm; upper right and bottom: 50 μm.

The better to determine the contribution of SVF CD14^+^ cells to Matrigel vasculature, we analyzed for expression of *Ulex europaeus*-I lectin (UEA-I), which is specific for human vascular endothelium. Immunofluorescence analysis of consecutive Matrigel sections against mCD31 and UEA-1 showed a virtually identical staining pattern (Figure 7A,B). These findings demonstrate that SVF CD14^+^ cells give rise to endothelial cells, but additionally, that Matrigel implants were vascularized by blood vessels formed both from host-derived endothelial cells and from SVF CD14^+^ human cells. Co-staining for αSMA, which identifies human and murine perivascular cells, showed that vessel structures were composed of endothelial cells (green) closely associated with perivascular αSMA^+^ cells (red), the typical structure of mature blood vessels. However, immunostained sections demonstrated that Matrigel implants also contained vascular tubelike structures composed of αSMA^+^ cells, but devoid of mCD31^+^ or UEA-1-positive endothelial cells (Figure 7), probably indicating the presence of immature blood vessels, as previously reported [23].

**Figure 8:**
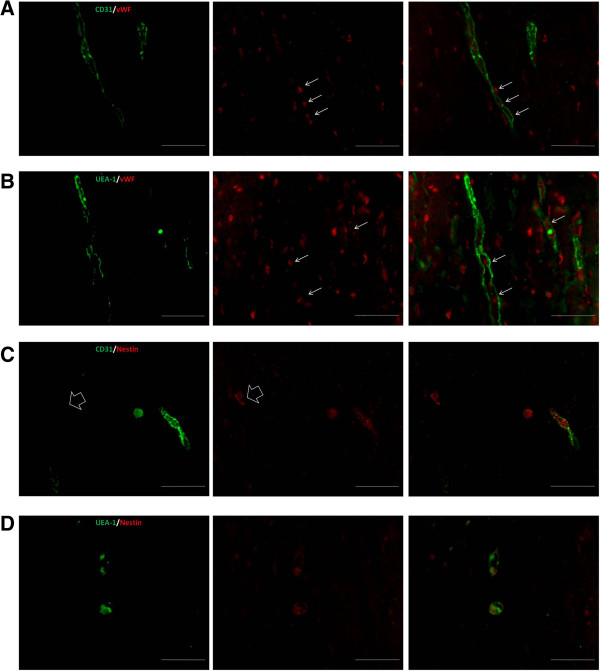
**Immunofluorescence detection of nestin in Matrigel implants.** Representative images of consecutive sections of Matrigel implants with SVF CD14^+^ cells stained by anti-mouse CD31 (green) and anti-vWF (red) **(A)**, and by UEA-1 (green) and anti-vWF (red) **(B)**. The arrows show colocalization of vWF-positive cells and endothelial cells of murine or human origin. However, in Matrigel implants, other vWF-positive cells not associated with blood vessels are shown. Double-immunofluorescence staining of human nestin (red) and mCD31 (green) **(C)**, and human nestin (red) and UEA-1 (green) **(D)**. In Matrigel implants, nestin was expressed in human and murine endothelial cells, although some nestin-positive cells (arrow) lacking mCD31 were observed. Scale bar, 50 μm.

Staining with anti-vWF to identify blood vessels of human and murine origin, demonstrated that Matrigel implants contained a significant number of vWF-positive cells, which were not associated with Matrigel vasculature (Figure 8A,B). Macrophages contribute to uptake of vWF [24] and are recruited into Matrigel implants supplemented with FGF-2 [25]. Here, we used nonsupplemented Matrigel implants and nude mice, but it is possible that FGF secreted by SVF CD14^+^ cells contributes to host-derived macrophage recruitment. Alternatively, vWF-positive cells could also identify SVF CD14^+^ cells contained in the implant.

Macrophages expressing the early pericyte marker NG2 proteoglycan have been shown to contribute to neovessel formation [23]. Therefore, to assess whether SVF CD14^+^ cells displayed human pericyte markers, we performed immunostaining of human nestin, which is expressed in pericytes [26] and also in endothelial cells of newly formed blood vessels [27]. Although the expression of nestin was very weak, this was restricted to endothelial cells (Figure 8C,D), and we did not observe nestin-positive cells in the perivascular area. However, single nestin-positive cells (indicated by arrow in Figure 8C) were observed.

## Discussion

Adipose tissue, similar to bone marrow, contains stromal cells that secrete multiple angiogenic factors [22], and delivery of these cells has angiogenic therapeutic potential [28]. Herein we demonstrated that the hematopoietic cell population contained in the SVF is highly enriched in CD45^+^CD14^+^ cells expressing CD34 antigen and an endothelium-like phenotype. Because CD34 is lost during leukocyte maturation, these CD14^+^CD34^+^ cells must represent a population of immature monocytes. Similarly, several groups have demonstrated the existence of a small subset of circulating CD14^+^ monocytes that exhibit stem/progenitor cell properties. Kuwana *et al.*[29] cultured CD14^+^ cells on fibronectin without angiogenic factors and identified a population of CD14^+^CD45^+^CD34^+^ monocytes with fibroblast-like morphology and the ability to differentiate into several mesodermal lineages. Kuwana *et al*. then demonstrated downregulation of CD14 and CD45 in this specific subset of monocytes when cultured in endothelial media; differentiation into endothelial cells expressing CD31, CD144, VEGFR1, VEGFR2, Tie-2, von Willebrand factor, e-NOS, and CD146; and vasculogenic properties *in vivo*[30]. Conversely, by using a highly sensitive antibody-conjugated magnetofluorescent liposomal technique, Romagnani *et al*. [31] demonstrated that a variable CD14^+^ cell proportion coexpressed CD34. These CD14^+^CD34^low^ cells, in contrast to fully differentiated CD14^+^CD34^-^ monocytes, exhibited high expression of the embryonic stem cell markers Nanog and Oct-4 and proliferated in the presence of early-acting hematopoietic cytokines. Because CD14^+^CD34^low^ staining identified the majority of circulating KDR^+^ cells that differentiated into mature endothelial cells [31], these cells may be the main source of PB-derived endothelial cells. However, CD45^+^CD34^+^ cells in human ovarian tumors reportedly share monocyte and endothelial cell phenotypic characteristics and also have the ability to generate blood vessels *in vivo*[32].

Our results show that SVF CD14^+^ cells induced a robust angiogenesis *in vivo* in the murine Matrigel plug assays, significantly higher than that induced by SVF-derived MSCs, and more important, they differentiated into fully functional endothelial cells, thereby contributing to Matrigel vasculature. Blood monocytes exit the circulation at a low frequency, migrate between vascular endothelial cells, and enter tissues. Among the several molecules involved in this process, PECAM-1/CD31 can drive the transmigration of a small subset of immature CD14^+^CD34^+^ monocytes by creating a haptotactic gradient [33] in the absence of chemokines [34]. Therefore, the physiological characteristics of adipose tissue [11] could favor recruitment of CD14^+^CD34^+^ monocytes, which are modulated toward an endothelium-like phenotype because they are in a highly angiogenic microenvironment [3,22]. According to this hypothesis, coculture experiments with circulating PB monocytes, together with SVF-derived MSCs, showed that MSCs promoted the acquisition of the endothelial markers KDR and Tie-2 in monocytes. But, importantly, these endothelium-like monocytes were highly angiogenic, and so the number of neovessels formed in implants containing these “transformed” monocytes was significantly increased when compared with those containing isolated PB monocytes or SVF-derived MSCs by approximately 6 or 2 times, respectively.

Since Asahara *et al*. [35] described the presence of circulating endothelial progenitor cells, many efforts have been devoted to identify and characterize these cells because they represent a potential therapeutic option for improving neovascularization [36]. However, many reports have indicated that these bone marrow-derived cells are mostly of myeloid origin [37]. Furthermore, in blood, VEGFR2 and/or Tie2 expression was mainly restricted to monocytes, and only cells expressing either of these antigens contributed to endothelialization of balloon-injured femoral arteries in mice [38]. It was recently demonstrated that common myeloid and granulocyte progenitor populations also have angiogenic properties [39]. Myeloid cells participate in physiological angiogenesis and in pathological processes [38,40-42], and in the cancer field, different myeloid cell populations have been shown to regulate tumor angiogenesis [43].

Although controversy still exists regarding whether myeloid cells are incorporated into newly formed vessels, elegant experiments by Grunewald *et al.*[44] demonstrated that VEGF alone was sufficient to recruit myeloid cells in specific organs, and once retained in the perivasculature, these cells induced *in situ* proliferation of resident endothelial cells through a repertoire of angiogenic activity.

Among the growth factors and cytokines with angiogenic activity, much attention has been paid to angiogenesis induced by VEGF and bFGF [23]. Results reported here show that these two angiogenic factors were highly secreted by SVF CD14^+^ cells, even in amounts significantly greater than those secreted by MSCs. Because HGF was highly secreted by MSCs, unlike what was observed in SVF CD14^+^ cells, HGF must play an important role in angiogenesis induced by MSCs. Although unanimity occurs on the angiogenic properties of G-CSF [45], some controversy exists about whether GM-CSF induces or inhibits angiogenesis [46]. However, when GM-CSF was administered together with G-CSF, angiogenesis was increased [47]. Therefore, it is possible that the secretion of G-CSF and GM-CSF by SVF CD14^+^ cells stimulates Matrigel angiogenesis.

Moreover, it was recently reported that Ang-2, a regulator of vessel maturation [48], can function as a vessel-destabilizing or as a proangiogenic molecule in endothelial cells, depending on whether endothelial cells express Tie2 or not [49]. However, from the experiments performed in this work, it is difficult to establish the role of Ang-2 in this *in vivo* angiogenesis assay.

Overall, our results show that SVF CD14^+^ cells differentiated into fully functional vascular endothelial cells, and promoted angiogenesis through a paracrine manner, suggesting that tissue-resident CD14^+^ monocytes must contribute to adipose tissue angiogenesis. Moreover, the results highlight the role of stroma in modulating the angiogenic capacity of monocytes recruited. Indeed, MSCs, in a paracrine manner, induced changes in both antigen expression and secretion ability of growth factors in PB monocytes, which could explain their higher angiogenic capacity.

SVF from human adipose tissue has emerged as a source of MSCs for regenerative medicine [40,50]. However, we show, as proof of concept, that angiogenesis induced by SVF CD14^+^ cells is superior to that induced by SVF-derived MSCs. We think these findings are relevant for novel angiogenic therapies based on the use of SVF CD14^+^ cells.

## Conclusions

The results from this study demonstrate that SVF from human adipose tissue contains CD14^+^ cells expressing CD34, CD31, KDR, Tie-2, and Flt-1. In the Matrigel-plug assay, these endothelium-like CD14^+^ cells secreted high amounts of angiogenic factors, induced angiogenesis more efficiently than SVF-derived MSCs, differentiated into endothelial cells, and contributed to Matrigel vasculature. Although angiogenesis is required in growing adipose tissue [9], the mechanisms of adipose tissue vascularization are not fully understood. The present data provide evidence that adipose tissue-resident monocytes might contribute to new vasculature. MSCs from adipose tissue are gaining interest for angiogenic therapies, as these cells are easily isolated. Our findings suggest that CD14^+^ cells isolated from the SVF may augment the efficacy of therapeutic angiogenesis induced by SVF-derived MSCs, and could potentially be used alone to promote tissue vascularization.

## Abbreviations

Ang-2: Angiopoietin-2; APC: allophycocyanin; APC-Cy7: allophycocyanin-cyanin7; bFGF: basic fibroblast growth factor; BSA: bovine serum albumin; DAB: 3, 3′-diaminobenzidine; EDTA: ethylendiaminetetraacetic acid; eNOS: endothelial nitric-oxide synthase; FBS: fetal bovine serum; FITC: fluorescein-isothiocyanate; G-CSF: granulocyte colony-stimulating factor; GM-CSF: granulocyte macrophage colony-stimulating factor; H&E: hematoxylin and eosin; HGF: hepatocyte growth factor; MSCs: mesenchymal stromal cells; PB: peripheral blood; PBS: phosphate-buffered saline; PE: phycoerythrin; PECAM-1: platelet/endothelial cell adhesion molecule-1; PE-Cy7: phycoerythrin-cyanin7; RT: room temperature; SVF: stromal vascular fraction; UEA-1: *Ulex europaeus* agglutinin 1; VEGF: vascular endothelial growth factor; VEGFR2: vascular endothelial growth factor receptor 2; vWF: von Willebrand factor; α-SMA: α-smooth muscle actin.

## Competing interests

The authors declare that they have no competing interests.

## Authors’ contributions

AN participated in all *in vitro* and *in vivo* experiments, including histologic analyses, and drafted and revised the manuscript. SM was responsible for the collection of samples of human adipose tissue, participated in all experiments involving animals, and revised the manuscript. NR participated in the isolation and culture of cell subsets, in the preparation of cells for flow cytometry, and revised the manuscript. FCU performed the analysis and interpretation of data from flow cytometry and drafted and revised the manuscript. MDM was responsible for the conception and design of the study, participated in all aspects of the study, including *in vitro* and *in vivo* experiments, performed the analysis and interpretation of data, and wrote the manuscript. All authors read and approved the final manuscript. All authors agree to be accountable for all aspects of the work in ensuring that questions related to the accuracy or integrity of any part of the work are appropriately investigated and resolved.
